# The impact of on-site cardiac surgical backup on clinical outcomes of acute coronary syndrome—analysis of the ACSIS national registry

**DOI:** 10.3389/fcvm.2023.1207473

**Published:** 2023-09-01

**Authors:** Gassan Moady, Tal Ovdat, Ronen Rubinshtein, Amnon Eitan, Elias Daud, Ziad Arow, Shaul Atar

**Affiliations:** ^1^ Department of Cardiology, Galilee Medical Center, Nahariya, Israel; ^2^ Azrieli Faculty of Medicine, Bar Ilan University, Safed, Israel; ^3^ The Israeli Center of Cardiovascular Research, Tel Hashomer, Israel; ^4^ Sackler Faculty of Medicine, Tel-Aviv University, Tel-Aviv, Israel; ^5^ Heart Institute, Edith Wolfson Medical Center, Holon, Israel; ^6^ Department of Cardiology, Carmel Medical Center, Haifa, Israel; ^7^ Department of Cardiology, Meir Medical Center, Kfar Saba, Israel

**Keywords:** acute coronary syndrome, percutaneous coronary intervention, cardiac surgery, acute myocardal infarct, cardiac care, CABG (Coronary artery bypass grafting surgery)

## Abstract

**Background:**

The availability of advanced technologies for mechanical support in hospitals with on-site cardiac surgery (CS), along with the ability to perform urgent coronary artery bypass graft (CABG) surgery, may result in improved clinical outcomes in patients with acute coronary syndrome (ACS).

**Methods:**

We conducted a retrospective analysis of the bi-annually Acute Coronary Syndrome Israeli Survey (ACSIS) registry from the year 2000 to 2020, performed in hospitals with and without CS. Mortality rates and major adverse cardiac and cerebrovascular events (MACCE) rates are reported. We evaluated two periods of the study—early (2000–2010) vs. late (2011–2020). Propensity score matching was performed to reduce bias between the two groups.

**Results:**

The study included 16,979 patients (52.3% in the on-site CS group). Patients in the on-site CS group were more likely to undergo percutaneous coronary intervention (PCI), (odds ratio [OR], 1.26 [95% CI, 1.18–1.35]; *p* < 0.001) and CABG [OR, 1.91 (95%CI, 1.63–2.24); *P* < 0.001], and patients in hospitals without on-site CS had higher 30-day MACCE [OR, 1.17 (95% CI, 1.07–1.27); *p* < 0.0005]. Overall, there was no difference in 1-year mortality (hazard ratio [HR], 0.98 [95% CI, 0.89–1.08]; *p* = 0.71) between the groups. During the late period of the study, patients in the group without on-site CS had lower 30-day mortality [OR, 0.69 (95% CI, 0.49–0.97); *P* = 0.04], yet with no difference in 1-year mortality [HR, 0.81 (95% CI, 0.65–1.01); *p* = 0.07].

**Conclusions:**

The availability of on-site CS resulted in variations in treatment modality, yet it did not affect the clinical outcomes of ACS. A trend to a better short-term outcomes was noted in hospitals without CS during the late period of the study, which warrants further investigation.

## 1. Introduction

Acute coronary syndrome (ACS) continues to be a leading cause of morbidity and mortality worldwide. The mainstay therapy of ACS is based on medications to limit ischemia along with coronary revascularization by percutaneous coronary intervention (PCI) or coronary artery bypass graft (CABG) (1–3). Routine use of radial approach together with advances in PCI techniques and new generation of drug-eluting stents (DES) had led to improvement in clinical outcomes overtime (4). Guideline-directed medical therapy, including wide-spread use of statins and antiplatelet drugs have also contributed to a significant reduction of 1-year mortality from 22% to 11% during the last two decades (5).

Previous studies showed that the outcome of elective and non-elective PCI in hospitals without on-site cardiac surgery (CS) is non-inferior to that in hospitals with on-site CS (6–14). Accordingly, on-site surgical backup is no longer mandatory for performing PCI. However, having on-site CS means more than just being able to perform urgent CABG when indicated. Advanced technologies for mechanical support such as extracorporeal membrane oxygenation (ECMO), left ventricular assist device (LVAD) or Impella device, may potentially improve clinical outcomes of mechanical or procedural complications and acute heart failure in patients with ACS. Therefore, the whole decision-making process in the management of ACS may be influenced by the availability of on-site CS.

The Acute Coronary Syndrome Israeli Survey (ACSIS) is a nationwide registry conducted every two years in all Israeli hospitals in patients hospitalized with ACS in cardiology departments and intensive cardiac care units since 2000. Since only half of the hospitals have on-site CS backup, we decided to assess differences and trends in outcomes of patients with ACS in centers with and without on-site CS (15).

## 2. Methods

### 2.1. Study subjects

The study is based on data from the ACSIS registry. The survey prospectively collects prespecified data on patients admitted with the diagnosis of ACS including unstable angina pectoris, non-ST-segment elevation myocardial infarction (NSTEMI) and ST-segment elevation myocardial infarction (STEMI) to Intensive Cardiac Care Units (ICCU) and cardiology departments in 26 public hospitals in Israel. All patients enrolled in the ACSIS registry between 2000 and 2020 were included in the study. The study was approved by the Institutional Review Board of each site. All patients signed an informed consent.

### 2.2. Data collection and definitions

During the survey, demographics, laboratory data, and clinical outcomes have been entered at each site on a standardized case report form. Follow-up information was collected by nurses and study-coordinators by telephone interview at 30 days. Data on 1-year survival were obtained from the Israeli National Population Registry. The diagnosis of myocardial infarction was determined by attending physicians based on a pattern of rise and/or fall of cardiac troponin with at least one value above the 99th percentile along with other clinical, electrocardiographic, and imaging modalities, consistent with the acceptable criteria for the diagnosis of myocardial infarction (16, 17). Obstructive coronary artery disease (CAD) was defined as stenosis of ≥70% in at least 1 of the main epicardial coronary arteries or its major branches by visual estimation. Chronic renal failure was defined as an estimated glomerular filtration rate (eGFR) of <60 ml/min/1.73 m^2^, calculated at baseline by the CKD-EPI equation.

### 2.3. Outcomes

Major adverse cardiac and cerebrovascular events (MACCE), 30-day mortality, and 1-year mortality rates were obtained. MACCE was defined as cardiovascular death, non-fatal myocardial infarction, cerebrovascular accident, and urgent revascularization. To identify trends, we evaluated the outcomes in the early (2000–2010) and late (2011–2020) periods of the study.

### 2.4. Statistical analysis

Patient characteristics are presented with percentages for categorical variables and mean ± standard deviation (SD) or median with interquartile range (IQR) for normal/non-normal distributed continuous variables (normality was assessed using Shapiro-Wilk test). The study groups were tested with chi-square for categorical variables and with t-test or Mann–Whitney–Wilcoxon test for normal/non-normal distributed continuous variables. To assess relationship between study groups and 1-year mortality, survival curves were presented, using Kaplan-Meier log rank test. To reduce bias between the groups, we performed a propensity score matching with the following variables: age, sex, dyslipidemia, hypertension, current smokers, diabetes mellitus (DM), family history of CAD, prior CABG, prior PCI, prior cerebrovascular accident (CVA) or transient ischemic attack (TIA), peripheral arterial disease (PAD), history of congestive heart failure (CHF), admission KILLIP class and primary diagnosis (STEMI/NSTEMI). A 1:1 matching was conducted with a 0.013 caliper (area under the curve of the model was 0.56), with total of 15,122 patients. Univariable models were performed in the matched cohort for the outcomes mentioned above. All tests were conducted at a two sided overall 5% significance level (α = 0.05). All analyses were performed using R (R-studio, V.4.0.3, Vienna, Austria).

## 3. Results

### 3.1. Patient characteristics

A total of 16,979 patients with ACS [median age, 64 years (IQR, 54–74 years); 77% male] were included in the study. Of them, 8,095 (47.7%) were treated in hospitals without on-site CS. The final diagnosis was STEMI in 45.4%, NSTEMI in 38.8%, and unstable angina pectoris (UAP) in 15.8%. Diagnostic coronary angiography was performed in 87.3% of the patients. Overall, 61% of the patients underwent PCI, 5.8% referred for CABG while conservative approach was adopted in 33.2%. Patients in the group without on-site CS were younger with high prevalence of tobacco use, high body mass index (BMI), DM, CHF, and a history of CVA/TIA. The baseline characteristics of the study population are presented in Table 1.

**Table 1:** Baseline characteristics of the study population.

	Overall	On-site CS	Without on-site CS	*P* value	Missing (%)
	16,979	8,884	8,095		
Age, Years [median (IQR)]	64 [54–74]	64 [55–74]	63 [55–74]	0.006	0
Male	13,112 (77.2%)	6,933 (78.0%)	6,179 (76.3%)	0.008	0
BMI (Kg/m^2^), [Mean (IQR)]^a^	27.04 [24.57,30.07]	26.83 [24.49,29.75]	27.18 [24.62,30.30]	0.001	28.2
Dyslipidemia	11,019 (65.2%)	5,767 (65.2%)	5,252 (65.3%)	0.925	0.4
Hypertension	10,091 (59.7%)	5,32,060.1%)	4,771 (59.2%)	0.247	0.7
Diabetes mellitus	6,123 (36.2)	3,145 (35.5)	2,978 (37.0)	0.044	0.3
Current smoker	6,330 (37.5)	3,142 (35.6)	3,188 (39.7)	<0.001	0.7
Family history of CAD	4,088 (26.0)	2,168 (26.2)	1,920 (25.8)	0.551	7.4
Chronic renal failure^b^	1,853 (11.0)	970 (10.9)	883 (11.0)	0.972	0.4
Prior MI	5,312 (31.4)	2,753 (31.1)	2,559 (31.7)	0.363	0.3
Prior PCI	4,792 (28.3)	2,585 (29.2)	2,207 (27.4)	0.011	0.4
Prior CABG	1,668 (9.9)	921 (10.4)	747 (9.3)	0.017	0.3
Prior CVA/TIA	1,383 (8.2)	665 (7.5)	718 (8.9)	0.001	0.4
History of heart failure	1,370 (8.1)	624 (7.0)	746 (9.3)	<0.001	0.4
PAD	1,405 (8.3)	765 (8.6)	640 (7.9)	0.113	0.4
Prior medications					
Aspirin	7,043 (47.9)	3,696 (47.3)	3,347 (48.5)	0.164	13.3
Antiplatelets^c^	1,362 (9.4)	743 (9.6)	619 (9.2)	0.419	14.6
Beta blockers	5,346 (37.2)	2,843 (37.4)	2,503 (37.1)	0.697	15.4
Statins	6,623 (47.4)	3,579 (48.5)	3044 (46.2)	0.006	17.8
ACEi	3,170 (31.2)	1,590 (29.5)	1,580 (33.2)	<0.001	40.2
ARB	1,218 (12.5)	705 (13.6)	513 (11.2)	<0.001	42.4
CCB	2,999 (21.6)	1,596 (21.6)	1,403 (21.5)	0.851	18.1
Diuretics	2,072 (17.5)	1,080 (17.0)	992 (18.0)	0.194	30.2
Transportation mode^d^					1
Private car	7,475 (44.5)	3,698 (42.0)	3,777 (47.2)	<0.001	
Mobile ICCU	6,217 (37.0)	3,514 (39.9)	2,703 (33.8)	<0.001	
Regular ambulance	2,210 (13.1)	997 (11.3)	1,213 (15.2)	<0.001	
Not relevant (inpatient)	913 (5.4)	605 (6.9)	308 (3.8)	<0.001	
Dx at arrival					0
STEMI	7,710 (45.4)	4,123 (46.4)	3,587 (44.3)	<0.001	
NSTEMI	6,587 (38.8)	3,470 (39.1)	3,117 (38.5)	<0.001	
UAP	2,682 (15.8)	1,291 (14.5)	1,391 (17.2)	<0.001	
KILLIP class^e^					2.4
I	13,963 (84.3)	7,293 (84.3)	6,670 (84.2)		
II	1,512 (9.1)	795 (9.2)	717 (9.1)		
III	814 (4.9)	387 (4.5)	427 (5.4)		
IV	280 (1.7)	176 (2.0)	104 (1.3)		
Heart rate (bpm) [median (IQR)]	78.00 [67.00, 91.00]	77.00 [67.00, 90.00]	79.00 [68.00, 92.00]	<0.001	1.8

ACE I, angiotensin converting enzyme inhibitors; ARB, angiotensin receptor blocker; BMI, body mass index; CABG, coronary artery bypass grafting; CCB, calcium channel blocker; CVA, cerebrovascular accident; ICCU, intensive cardiac care unit; CAD, coronary artery disease; IQR, Interquartile range; PAD, peripheral arterial disease; PCI, percutaneous coronary intervention; SD, standard deviation; STEMI, ST-segment elevation myocardial infarction; TIA, transient ischemic attack; UAP, unstable angina pectoris.

^a^ Calculated as weight in kilograms divided by height in meters squared.

^b^ Defined as estimated glomerular filtration rate less than 60 ml/min/ 1.73 m^2.^

^c^ Including either clopidogrel, ticagrelor, or prasugrel.

^d^ The facility used to transfer the patient to the emergency room.

^e^ Applied in cases of myocardial infarction as: I, no clinical signs of heart failure; II, signs of mild congestion like rales, S3 gallop, or jugular venous distention; III, frank pulmonary edema; IV, cardiogenic shock.

During the index hospitalization, patients in the on-site CS group were more likely to undergo PCI regardless of the category of ACS (STEMI/NSTEMI/UAP) and had higher rate of bleeding and blood transfusions (Table 2).

**Table 2:** Clinical course and treatment strategy during the index hospitalization.

*n*	Overall	On-site CS	Without on-site CS	*P* value	Missing (%)
	16,979	8,884	8,095		
Heart rate (bpm) [median (IQR)]	78.00 [67.00, 91.00]	77.00 [67.00, 90.00]	79.00 [68.00, 92.00]	<0.001	1.8
SBP	140.00 [123.00, 160.00]	140.00 [122.00, 160.00]	140.00 [123.00, 160.00]	0.372	1.7
KILLIP class^a^					2.4
I	13,963 (84.3)	7,293 (84.3)	6,670 (84.2)		
II	1,512 (9.1)	795 (9.2)	717 (9.1)		
III	814 (4.9)	387 (4.5)	427 (5.4)		
IV	280 (1.7)	176 (2.0)	104 (1.3)		
EF < 40%	2,992 (20.5)	1,511 (19.8)	1,481 (21.3)	0.028	14.2
LDL cholesterol (mg/dl) [median (IQR)]	106.0 [81.0,134.0]	105.0[80.0,132.0]	107.0 [81.0,135.0]	0.014	36.3
HDL cholesterol (mg/dl) [median (IQR)]	39.0 [32.0,46.0]	39.0 [33.0,46.0]	38.0 [32.0,46.0]	0.001	34.4
Creatinine (mg/dl) [median (IQR)]	1.0 [0.86,1.22]	1.0 [0.86,1.24]	1.0 [0.86,1.2]	0.071	14.3
Hemoglobin (g/dl) [median (IQR)]	13.7 [12.4,14.9]	13.7 [12.4–14.9]	13.7 [12.4,14.9]	0.549	12.9
Acute renal failure^b^	1,018 (6.0)	543 (6.1)	475 (5.9)	0.556	0.4
Bleeding	241 (1.4)	152 (1.7)	89 (1.1)	0.001	0.2
Blood transfusion	172 (3.1)	107 (3.6)	65 (2.5)	0.025	67.2
Coronary angiography	11,470 (87.3)	6,079 (88.4)	5,391 (86.0)	<0.001	0.0
PCI	10,352 (61.0)	5,656 (63.7)	4,696 (58.0)	<0.001	22
Time to PCI (minutes) (median) [IQR]^c^	195.0 [132.0,331.0]	200.0 [135.0,338.5]	188.0 [130.0,330.0]	0.094	18.2
CABG^d^	724 (5.8)	456 (6.9)	268 (4.5)	<0.001	25
ACUTE CVA/TIA	105 (0.6)	55 (0.6)	50 (0.6)	1.0	0.3
VSR	21 (0.1)	12 (0.1)	9 (0.1)	0.828	0.3
Hemodynamically significant RVI^e^	87 (0.8)	47 (0.8)	40 (0.8)	0.891	35.0
Stent thrombosis^f^	80 (0.7)	45 (0.8)	35 (0.7)	0.601	35.1
Primary VF	314 (1.9)	168 (1.9)	146 (1.8)	0.733	0.2
AV block (2d/3d)	388 (2.3)	223 (2.5)	165 (2.0)	0.049	0.3
New-onset AF	901 (5.3)	489 (5.5)	412 (5.1)	0.258	0.2
Asystole	365 (2.2)	182 (2.1)	183 (2.3)	0.354	0.2
Pericarditis	108 (0.6)	52 (0.6)	56 (0.7)	0.43	0.2

ACE, angiotensin converting enzyme; AF, atrial fibrillation; ARB, angiotensin receptor blocker; AV block, atrioventricular block; CABG, coronary artery bypass grafting; CVA, cerebrovascular accident; EF, ejection fraction; HDL, high-density lipoprotein; LDL, low-density lipoprotein; RVI, right ventricular infarction; SBP, systolic blood pressure; TIA, transient ischemic attack; PCI, percutaneous coronary intervention; VF, ventricular fibrillation; VSR, ventricular septal rupture.

^a^ Applied in cases of myocardial infarction as: I, no clinical signs of heart failure; II, signs of mild congestion like rales, S3 gallop, or jugular venous distention; III, frank pulmonary edema; IV, cardiogenic shock.

^b^ Defined as increase in serum creatinine by ≥0.3 mg/dl or a percentage increase of more than 50%.

^c^ refers to time from symptom onset to wire-crossing in STEMI.

^d^ In the no on-site CS group, the number refers to patients who were transferred to hospitals with on-site surgical backup.

^e^ When diagnosis was confirmed by echocardiography and ECG changes associated with hypotension requiring intravenous fluids or inotropic support.

^f^ Definite, probable, or possible.

The baseline characteristics and clinical course in the matched cohort are provided in the Supplementary Material.

In Table 3 we summarize the medical therapy and recommendation for cardiac rehabilitation at discharge and 30 days post ACS.

**Table 3:** Medications at discharge and at 30-day follow-up.

Treatment at discharge					
	Overall	With CS	Without CS	*P* value	Missing (%)
Aspirin	15,635 (94.4)	8,226 (94.9)	7,409 (93.8)	0.001	2.4
Other antiplatelet^a^	11,962 (72.8)	6,412 (74.5)	5,550 (71.1)	<0.001	3.3
Statins	14,005 (85.2)	7,349 (85.5)	6,656 (84.9)	0.275	3.2
ACEi/ARB	12,075 (74.1)	6,527 (76.5)	5,548 (71.4)	<0.001	4.0
Beta blockers	12,925 (79.8)	6,941 (81.8)	5,984 (77.7)	<0.001	4.7
30-day follow-up					
Cardiac rehabilitation^b^	4,681 (49.5)	2,778 (56.9)	1,903 (41.7)	<0.001	44.3
Aspirin	7,084 (95.6)	3,689 (95.8)	3,395 (95.4)	0.402	56.4
Other antiplatelet	4,050 (61.2)	2,093 (60.0)	1,957 (62.5)	0.039	61.0
Statins	7,074 (95.4)	3,645 (95.4)	3,429 (95.4)	0.982	56.3
ACEi	4,643 (67.5)	2,443 (67.8)	2,200 (67.2)	0.609	59.5
ARBs	923 (14.6)	511 (15.2)	412 (13.9)	0.148	62.7
Beta Blockers	5,880 (82.7)	3,120 (84.1)	2,760 (81.2)	0.001	58.1

ACEi, angiotensin converting enzyme inhibitors; ARB, angiotensin receptor blocker.

^a^ Including either clopidogrel, ticagrelor, or prasugrel.

^b^ Referred to patient referral to the rehabilitation program not to the actual participation.

### 3.3. Thirty-day and 1-year outcomes

**Figure 1:**
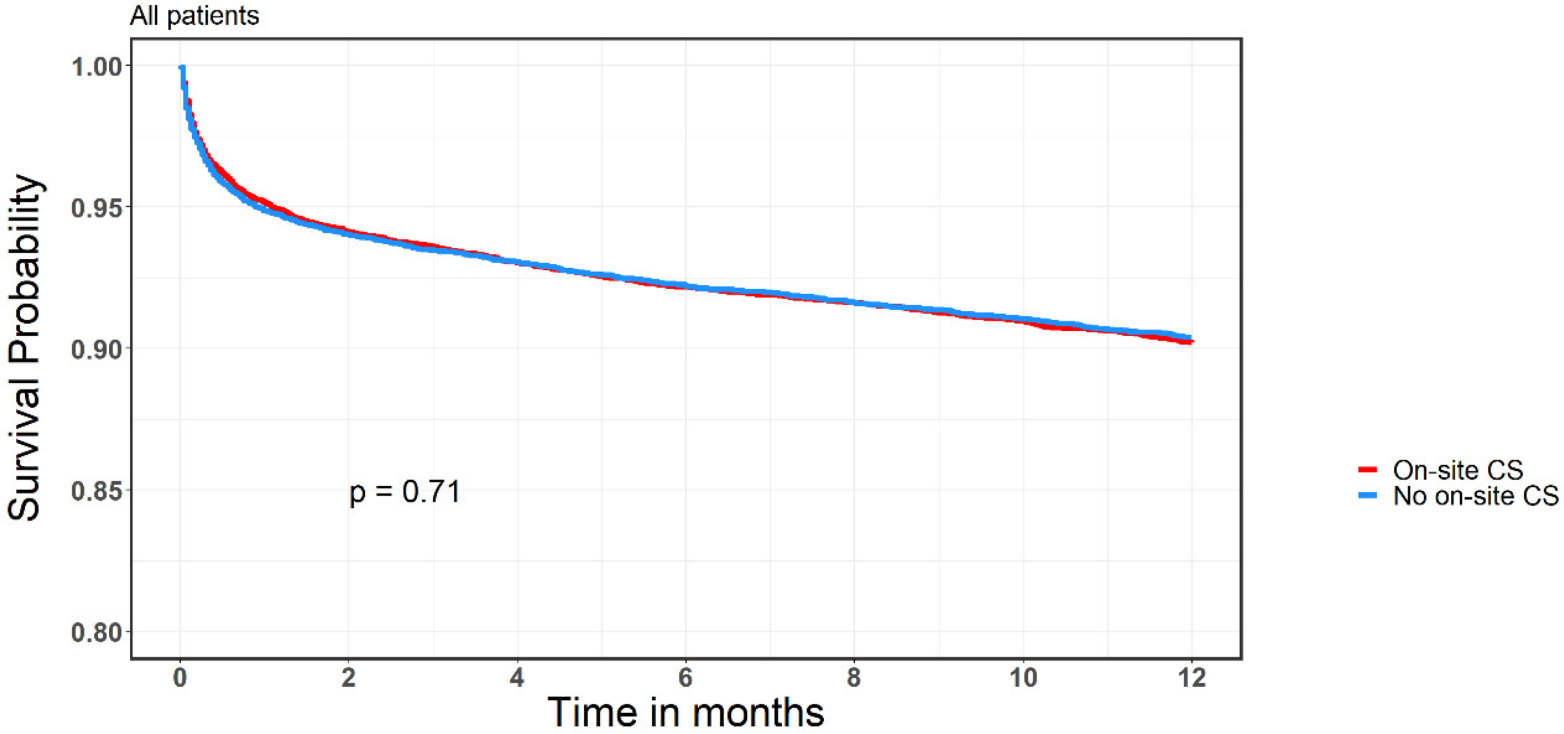
Kaplan-Meier survival curve, onsite and non-onsite CS groups.

**Figure 2:**
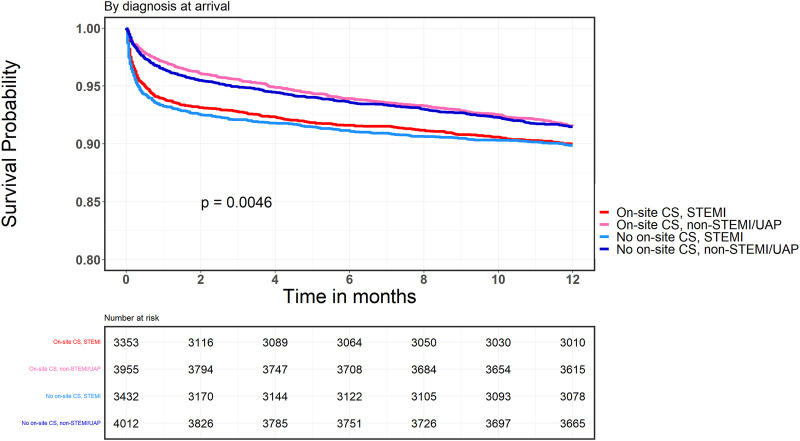
Kaplan-Meier survival curve, onsite and non-onsite CS groups according to clinical presentation and presence or absence of CS.

The 30-days MACCE rate was higher in the group without on-site CS [OR, 1.17 (95% CI, 1.07–1.27); *p* < 0.0005]. However, there was no difference in mortality rates after one month [OR, 1.08 (95% CI, 0.94–1.24); *p* = 0.3] or one year [OR, 0.98 (95% CI, 0.89–1.08); *p* = 0.71]. Table 4 presents the breakdown of 30-day MACCE between CS and no-CS groups. Only “urgent revascularization” (due to recurrent angina) was significantly higher in the group without on-site CS, with no other differences in hard outcomes. Survival curves of the study population are given in Figure 1. Survival curve stratified by diagnosis on arrival (STEMI vs. NSTEMI/UAP) is presented in Figure 2.

**Table 4:** Study outcomes (30-days MACCE).

	Study cohort (16,979)	With	Without	*P*
		on-site CS (8,884)	on-site CS (8,095)	
MACCE	2,402 (14.1)	1,176 (13.3)	1,226 (15.2)	<0.001
All-cause mortality	822 (4.8)	415 (4.7)	407 (5.1)	0.31
Non-fatal MI	286 (1.9)	144 (1.9)	142 (2.0)	0.59
Stroke	82 (0.5)	45 (0.6)	37 (0.5)	0.73
Recurrent angina	798 (4.7)	358 (4.0)	440 (5.5)	<0.001
Stent thrombosis^a^	84 (1.2)	46 (1.2)	38 (1.1)	0.83
Urgent PCI	178 (1.2)	86 (1.1)	92 (1.3)	0.319
Urgent CABG^b^	183 (1.2)	65 (0.8)	118 (1.7)	<0.001

^a^ Defined as definite, probable, or possible.

^b^ In centers without on-site CS, this refers to patients transferred urgently to a center with CS for surgical revascularization.

CABG, coronary artery bypass grafting; MACCE, major adverse cardiac and cerebrovascular events; MI, myocardial infarction; PCI, percutaneous coronary intervention.

CABG, coronary artery bypass graft; CI, confidence interval; MACCE, major adverse cardiac and cerebrovascular events; OR, odds ratio; PCI, percutaneous coronary intervention.

### 3.4. Early vs. late periods

In order to identify trends in outcomes in the matched cohort, we also evaluated two periods of the study, early (2000–2010) and late (2011–2020). Overtime, the percentage of patients treated by PCI increased in the two groups and was similar during the late period [OR on-site vs. without on-site CS, 1.07 (95% CI, 0.94–1.21); *p* = 0.31]. Referral to CABG was also similar in the two groups during the late period [OR, 1.22 (95% CI, 0.9–1.66); *p* = 0.2]. Bleeding events have also decreased in both groups [OR, 1.57 (95% CI, 0.99–2.53); *p* = 0.07]. Surprisingly, the group without on-site CS had lower 30-day mortality [OR, 0.69 (95% CI, 0.49–0.97); *p* = 0.04]. Yet, there was no difference in 1-year mortality rate [HR, 0.81 (95% CI, 0.65–1.01); *p* = 0.07]. The main outcomes of the study population are summarized in Table 5. Using multivariate analysis, 30-days mortality was not different between the groups [HR 0.95, (95% CI 0.81–1.13); *p* = 0.59]. Yet, 1-year mortality was still lower in hospitals without on-site CS, albeit not significantly [HR 0.91, (95% CI 0.81–1.01); *p* = 0.08]. The 30-days MACCE was significantly higher in those sites [HR 1.12 (95% CI 1.01–1.24), *p* = 0.03].

**Table 5:** Summary of outcomes in the complete period (A), and during the late period (B).

A. Complete study period	PCI	CABG	Bleeding	MACCE	30-day mortality	1-year mortality
With vs. Without on-site CS						
OR with 95% CI	1.26 (1.18, 1.35)	1.91 (1.63, 2.24)	1.53 (1.15, 2.05)	0.85 (0.79, 0.94)	0.93 (0.8–1.06)	0.98 (0.89- 1.08)
	*P* < 0.001	*P* < 0.001	*P* = 0.004	*P* = 0.0005	*P* = 0.3	*P* = 0.71
B. Late period						
	1.04 (0.91, 1.17)	1.23 (0.91, 1.67)	1.49 (0.95, 2.39)	1.16 (0.94, 1.43)	1.45 (1.03, 2.04)	1.23 (0.99, 1.54)
	*P* = 0.59	*P* = 0.19	*P* = 0.10	*P* = 0.16	*P* = 0.04	*P* = 0.07

CABG, coronary artery bypass graft; CI, confidence interval; MACCE, major adverse cardiac and cerebrovascular events; OR, odds ratio; PCI, percutaneous coronary intervention.

OR are presented as with vs. without on-site CS.

## 4. Discussion

In this nationwide study, we found that having an on-site CS may alter decision-making with respect to treatment strategy but does not affect 1-year outcomes in patients with ACS. To our knowledge, this is the largest study aimed to investigate the differences in clinical outcomes of patients with ACS (including those who were medically managed) in hospitals with and without on-site CS backup. In contrast to other large registry studies, such as the ORPKI registry (14), most of the studies evaluated only the outcomes of primary PCI in centers with and without on-site CS.

Since an invasive approach (PCI or referral to CABG) was more prevalent in the on-site CS group, we assumed that decision-making by the treating physician may be affected by the availability of on-site CS. The dilemma in decision-making may predominantly be crucial in special populations such as the elderly and those with complex coronary anatomy. Elderly patients with severe comorbidities and poor functional capacity are often offered medical therapy rather than PCI to avoid potential PCI-related complications and the need for emergent transfer for surgical treatment. Patients with complex coronary anatomy in hospital without on-site CS may be treated more often by PCI rather than CABG due to the lack of on-site heart-team collaborative decisions (18–20). In hospitals without on-site CS, the potential paucity of various mechanical support devices (such as ECMO, LVAD) may limit treatment options and alter decision-making in complex cases or when a mechanical complication occurs. The disparity in medical support devices is further complicated by the baseline severe co-morbidities of patients in certain low-income regions. The inaccessibility to medical facilities in those low-income regions, along with poor awareness of the patients to their medical condition may explain some of these differences (21, 22). In our study population, patients in the group without on-site CS had higher prevalence of obesity, diabetes, history of CVA/TIA, tobacco use and CHF. Also, they were more likely to be admitted to the hospital by private vehicle rather than mobile ICCU units. Bleeding events were more common in the on-site CS group, and more blood transfusions were needed, albeit it was not translated to higher mortality rates. We assume that most bleeding events and blood transfusions in this group are mostly CABG-related, yet the data on bleeding sites is not available.

In the short term, a higher rate of 30-day MACCE and readmissions were observed in the group without on-site CS. One of the important predictors of clinical outcomes in ACS is the quality of medical therapy and the adherence to medications prescribed at discharge (23, 24). In addition, patients in the group without on-site CS were less likely to be referred to rehabilitation programs, which have also been proven to reduce mortality after ACS (25).

Despite the variations in invasive strategy, there was no difference in 1-year mortality between the groups. These results were consistent after propensity score matching and when the study population was stratified by ACS category (STEMI vs. NSTEMI). The analysis of clinical outcomes in the two periods (early vs. Late) aimed to reflect the changes in therapeutic approach, advances in techniques, and the cumulative experience of the medical staff. We observed an increase in PCI in the two groups overtime along with a decrease in the rate of MACCE and 1-year mortality. The consistent increase in PCIs overtime is contributed mainly to referring more elderly patients and patients with unprotected left main coronary artery disease or multivessel coronary disease to an invasive approach (26–30).

An unexpected finding of higher 30-day mortality was observed in the group with on-site CS during the late period. This may be related to the overall decrease in the volume of CABG operations (since PCI is now being performed in patients with more complex anatomy), possibly leading to lower expertise of cardiac surgeons and related CS staff (31).

Regardless of the trend in the late period, our findings reinforce the non-inferiority of hospitals without on-site CS backup in managing ACS. In accordance with our unexpected results, Noaman et al. described the outcomes of patients with ACS complicated by cardiogenic shock treated at hospitals with on-site CS compared to hospitals without on-site CS and found no difference in in-hospital MACCE and mortality rates (32). Since there was no difference in the mortality rate between the two groups, we conclude that the quality of decision-making process was not affected by the availability of CS on-site. Nevertheless, we cannot determine what was driving the higher 30-day mortality in the late period in the on-site CS group—either CABG-related death or PCI-related death, non-cardiac death or heart failure related death.

There was a discrepancy between guideline-recommended therapies vs. real world clinical practice, ie. more patients in the non-CS group were supposedly to receive medical therapy, but in actual fact, they were less likely to be discharged on optimal pharmacotherapy. Yet, as we look the 30 days treatments (Table 3) we can see that there is no significant difference between the groups (except for a lower use of beta blockers). Therefore. we do not believe that this is a main contributor to our unexpected findings.

### Limitations

Our study carries some limitations. The study is not randomized and thus carries the limitations of a retrospective analysis. Yet, it reflects a non-biased real-world data. Therefore, we performed a propensity score matching to reduce selection bias. The study lacks long-term follow-up of cardiovascular events beyond 1 year. We do not provide coronary anatomy complexity data (such as using SYNTAX score). Yet, we provided the crude results in real world population.

## Conclusion

Our study emphasizes the non-inferiority of hospitals without on-site CS backup in managing patients with ACS. The absence of on-site CS led to variations in treatment modality but did not jeopardize the outcomes of the patients. Furthermore, a trend of better short-term outcomes was noticed in the group without on-site CS during the late period of the study, which warrants further investigation.

## Data Availability

The original contributions presented in the study are included in the article/**Supplementary Material**, further inquiries can be directed to the corresponding author.
