# Supplementary material for: The impact of on-site cardiac surgical backup on clinical outcomes of acute coronary syndrome—analysis of the ACSIS national registry
Source: Front Cardiovasc Med. 2023 Sep 1;10:1207473. doi: 10.3389/fcvm.2023.1207473 (PMC10505675; doi:10.3389/fcvm.2023.1207473)
Supplement: Supplementary file 1 [file Datasheet1.pdf]

Supplementary

Non- standard Abbreviations and Acronyms

ACS-Acute Coronary Syndrome;

ACSIS-Acute Coronary Syndrome Israeli Survey

BMI-Body Mass Index

CABG-Coronary Artery Bypass Grafting;

CAD-Coronary Artery Disease;

CHF-Congestive Heart Failure;

CS-Cardiac Surgery;

CVA-Cerebrovascular Accident; TIA-Transient Ischemic Attack

eGFR-estimated Glomerular Filtration Rate

GRACE-Global Registry of Acute Coronary Events;

ICCU-Intensive Cardiac Care Unit;

IQR-Interquartile Range;

MACCE-Major Adverse Cardiac and Cerebrovascular Events;

MDRD-Modification of Diet in Renal Disease;

NSTEMI-Non-ST segment Elevation Myocardial Infarction;

PCI-Percutaneous Coronary Intervention

SD-Standard Deviation;

STEMI-ST segment Elevation Myocardial Infarction;

Table 1. Baseline demographic and clinical characteristics in the matched cohort.

	Overall	On-site CS	Without on-site CS	P value
	15122	7561	7561	
**Age, Years (median [IQR])**	63.00 [54.00, 73.00]	64.00 [54.00, 73.00]	63.00 [54.00, 74.00]	0.484
**Male**	11690 (77.3%)	5856 (77.5%)	5834(77.2%)	0.683
**BMI (Kg/m^2), (Mean [IQR])[a]**	27.04 [24.57, 30.04]	26.83 [24.49,27.17]	29.73 [24.62,30.19]	0.002
**Dyslipidemia**	9728 (64.7)	4885 (64.8)	4843 (64.5)	0.632
**Hypertension**	8864 (58.8)	4448 (59.0)	4416 (58.7)	0.699
**Diabetes mellitus**	5405 (35.9)	2716 (36.0)	2689 (35.7)	0.782
**Current smoker**	5788 (38.5)	2904 (38.6)	2884 (38.4)	0.830
**Family history of CAD**	3641 (25.9)	1826 (25.9)	1815 (25.9)	0.950
**Chronic renal failure[b]**	1606 (10.7)	816 (10.8)	790 (10.5)	0.571
**Prior MI**	4609 (30.6)	2273 (30.1)	2336 (31.0)	0.239
**Prior PCI**	4138 (27.5)	2096 (27.8)	2042 (27.1)	0.375
**Prior CABG**	1418 (9.4)	717 (9.5)	701 (9.3)	0.738
**Prior CVA/TIA**	1189 (7.9)	603 (8.0)	586 (7.8)	0.689
**History of heart failure**	1107 (7.3)	570 (7.5)	537 (7.1)	0.357
**PAD**	1200 (8.0)	598 (7.9)	602 (8.0)	0.879
**Prior medications**				
**Aspirin**	6187 (47.2)	3099 (46.5)	3088 (47.9)	0.117
**Other antiplatelet[c]**	1148 (8.9)	615 (9.3)	533 (8.4)	0.086
**Beta blockers**	4646 (36.3)	2365 (36.5)	2281 (36.1)	0.721
**Statins**	5782 (46.5)	3018 (48.0)	2764 (44.9)	<0.001
**ACE inh**	2774 (30.9)	1347 (29.2)	1427 (32.6)	0.001
**ARB**	1062 (12.2)	601 (13.5)	461 (10.9)	<0.001
**CCB**	2623 (21.1)	1326 (21.1)	1297 (21.1)	1.00
**Diuretics**	1793 (17.0)	911 (16.8)	882 (17.1)	0.708
**Transportation mode[d]**				<0.001
**Private car**	6698 (44.8)	3188 (42.5)	3510 (47.0)	
**Mobile ICCU**	5511 (36.8)	2975 (39.7)	2536 (34.0)	
**Regular ambulance**	1976 (13.2)	842 (11.2)	1134 (15.2)	
**Not relevant (inpatient)**	782 (5.2)	495 (6.6)	287 (3.8)	

**Diagnosis at arrival**				0.592
**STEMI**	6936 (45.9)	3450 (45.6)	3486 (46.1)	
**NSTEMI**	5840 (38.6)	2950 (39.0)	2890 (38.2)	
**UAP**	2346 (15.5)	1161 (15.4)	1185 (15.7)	

Abbreviations: ACE inh, angiotensin- converting enzyme inhibitors; ARB, angiotensin receptor blocker; BMI, body mass index; CABG, coronary artery bypass grafting; CCB, calcium channel blocker; CVA, cerebrovascular accident; ICCU, intensive cardiac care unit; CAD, coronary artery disease; IQR, Interquartile range; PAD, peripheral arterial disease; PCI, percutaneous coronary intervention; SD, standard deviation; STEMI, ST-segment elevation myocardial infarction; TIA, transient ischemic attack; UAP, unstable angina pectoris.

[a] calculated as weight in kilograms divided by height in meters squared.

[b] Defined as estimated glomerular filtration rate less than 60 ml/min/ 1.73m^2

[c] Including either clopidogrel, ticagrelor, or prasugrel.

[d] The facility used to transfer the patient to the emergency room.

Table 2. Summary of clinical course and treatment strategy during the index hospitalization (in the matched cohort).

	Overall	On-site CS	Without on-site CS	P value	Missing
	**15122**	**7561**	**7561**		
**Heart rate (bpm) (median [IQR])**	78.00 [67.00, 90.00]	78.00 [67.00, 90.00]	79.00 [68.00, 91.00]	0.001	1.8
**SBP**	140.00 [123.00, 160.00]	140.00 [122.00, 160.00]	140.00 [123.00, 160.00]	0.896	1.6
**KILLIP class [a]**				0.896	2.3
**I**	12570 (85.1)	6271 (85.2)	6299 (84.9)		
**II**	1307 (8.8)	645 (8.8)	662 (8.9)		
**III**	701 (4.7)	342 (4.6)	359 (4.8)		
**IV**	195 (1.3)	100 (1.4)	95 (1.3)		
**EF<40%**	2610 (20.2)	1257 (19.4)	1353 (21.0)	0.023	14.5
**LDL cholesterol (mg/dl) (median [IQR])**	107.00 [81.06, 134.00]	106.00 [81.00, 133.00]	108.00 [82.00, 136.00]	0.009	36.5
**HDL cholesterol (mg/dl) (median [IQR])**	39.00 [32.00, 46.00]	39.00 [33.00, 46.00]	38.00 [32.00, 46.00]	0.013	34.6
**Creatinine (mg/dl) (median [IQR])**	1.00 [0.86, 1.21]	1.00 [0.85, 1.22]	1.00 [0.86, 1.20]	0.936	14.6
**Hemoglobin (g/dl) (median [IQR])**	13.70 [12.40, 14.90]	13.70 [12.40, 14.90]	13.70 [12.40, 14.90]	0.337	13.0
**Acute renal failure[b]**	874 (5.8)	434 (5.8)	440 (5.9)	0.824	0.4
**Bleeding**	197 (1.3)	119 (1.6)	78 (1.0)	0.004	0.2
**Blood transfusion**	142 (3.0)	85 (3.3)	57 (2.6)	0.025	68.5
**Coronary angiography**	12311 (81.4)	6398 (84.6)	5913 (78.2)	<0.001	0.0
**PCI**	9247 (61.1)	4833 (63.9)	4414 (58.4)	<0.001	0.0
**Time to PCI (minutes) (median) [IQR][c]**	191.50 [130.00, 330.00]	195.00 [135.00, 330.00]	187.00 [130.00, 330.00]	0.178	0.0
**CABG[d]**	689 (4.6)	448 (5.9)	241 (3.2)	<0.001	0.3
**ACUTE CVA/TIA**	89 (0.6)	42 (0.6)	47 (0.6)	0.660	0.3

**VSR**	18 (0.1)	10 (0.1)	8 (0.1)	0.819	0.3
**Hemodynamically significant RVI**[e]	80 (0.8)	42 (0.8)	38 (0.8)	0.903	36.0
**Stent thrombosis**[f]	70 (0.7)	38 (0.8)	32 (0.7)	0.689	36.1
**Primary VF**	280 (1.9)	143 (1.9)	137 (1.8)	0.784	0.2
**AV block (2d/3d)**	84 (1.0)	41 (1.0)	43 (1.1)	0.613	0.3
**New-onset AF**	792 (5.2)	400 (5.3)	392 (5.2)	0.830	0.2
**Asystole**	322 (2.1)	147 (1.9)	175 (2.3)	0.121	0.2
**Pericarditis**	100 (0.7)	45 (0.6)	55 (0.7)	0.358	0.2

Abbreviations: ACE, angiotensin converting enzyme; AF, atrial fibrillation; ARB, angiotensin receptor blocker; AV block, atrioventricular block; CABG, coronary artery bypass grafting; CVA, cerebrovascular accident; EF, ejection fraction; HDL, high-density lipoprotein; LDL, low-density lipoprotein; RVI, right ventricular infarction; SBP, systolic blood pressure; TIA, transient ischemic attack; PCI, percutaneous coronary intervention; VF, ventricular fibrillation; VSR, ventricular septal rupture.

[a] Applied in cases of myocardial infarction as: I, no clinical signs of heart failure; II, signs of mild congestion like rales, S3 gallop, or jugular venous distention; III, frank pulmonary edema; IV, cardiogenic shock.

[b] Defined as increase in serum creatinine by $\geq$0.3 mg/dl or a percentage increase of more than 50%.

[c] Refers to time from symptom onset to wire-crossing in STEMI.

[d]In the no on-site CS group, the number refers to patients who were transferred to hospitals with on-site surgical backup.

[e] When diagnosis was confirmed by echocardiography and ECG changes associated with hypotension requiring intravenous fluids or inotropic support.

[f] Definite, probable, or possible.

Table 3. Medications at discharge and at 30-day follow up (matched cohort).

					**Treatment at discharge**
Aspirin	13970 (94.5)	7033 (95.2)	6937 (93.9)	0.001	2.3
Other antiplatelet	10623 (72.5)	5485 (74.7)	5138 (70.4)	<0.001	3.1
Statins	12461 (85.0)	6283 (85.7)	6178 (84.3)	0.019	3.1
ACE inh/ARB	10755 (73.8)	5558 (76.4)	5197 (71.3)	<0.001	3.7
Beta blockers	11549 (79.9)	5943 (82.1)	5606 (77.7)	<0.001	4.4
					**30-day follow-up**
Cardiac rehabilitation	4139 (49.8)	2378 (57.1)	1761 (42.5)	<0.001	45.0
Aspirin	6209 (95.7)	3161 (95.9)	3048 (95.5)	0.518	57.1
Other antiplatelet	3576 (61.4)	1802 (60.3)	1774 (62.6)	0.083	61.5
Statins	6171 (95.3)	3115 (95.3)	3056 (95.2)	0.917	57.2
ACE inh	4091 (67.7)	2095 (67.9)	1996 (67.6)	0.828	60.1
ARB	800 (14.3)	435 (15.1)	365 (13.5)	0.086	63.0
Beta Blockers	5148 (82.6)	2671 (84.0)	2477 (81.2)	0.004	58.8

ACE i, angiotensin converting enzyme inhibitors; ARB, angiotensin receptor blocker.

[a] Including either clopidogrel, ticagrelor, or prasugrel.

[b] Referred to patient referral to the rehabilitation program, and not to the actual participation.

.

Figure 1. Kaplan-Meier curves in the matched cohort

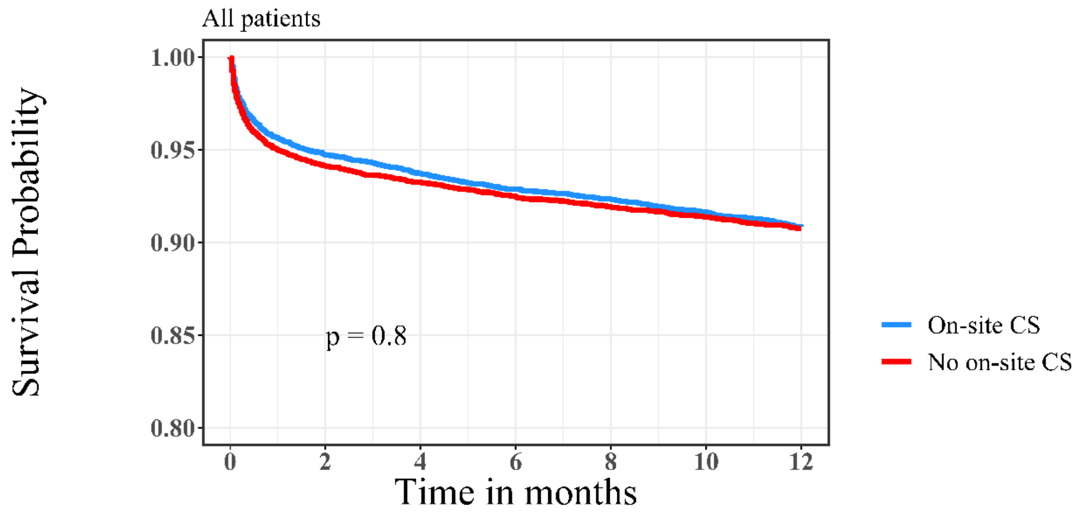

Number at risk

On-site CS	7308	6910	6836	6772	6734	6684	6625
No on-site CS	7444	6996	6929	6873	6831	6790	6743

Figure 2. Kaplan-Meier curves in the matched cohort stratified by diagnosis

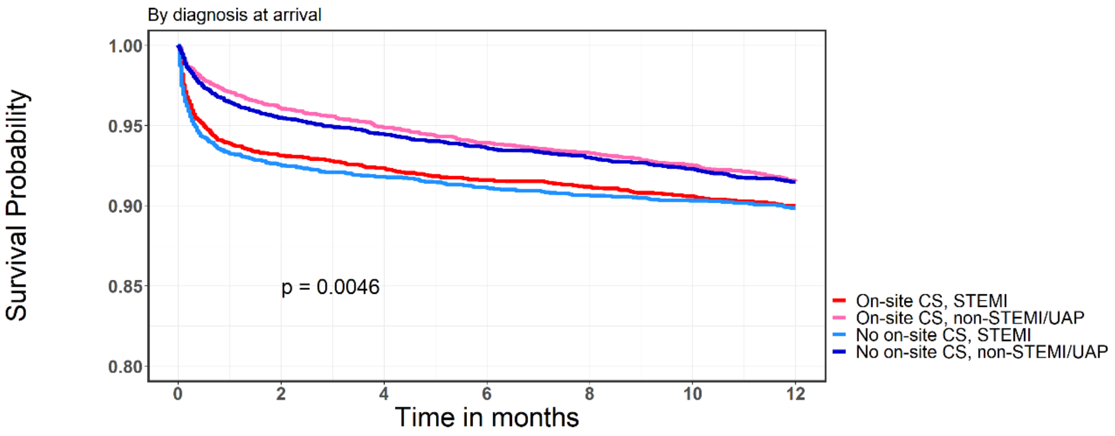

Number at risk

	0	2	4	6	8	10	12
On-site CS, STEMI	3353	3116	3089	3064	3050	3030	3010
On-site CS, non-STEMI/UAP	3955	3794	3747	3708	3684	3654	3615
No on-site CS, STEMI	3432	3170	3144	3122	3105	3093	3078
No on-site CS, non-STEMI/UAP	4012	3826	3785	3751	3726	3697	3665
